# Impact of transportation, storage, and retail shelf conditions on lettuce quality and phytonutrients losses in the supply chain

**DOI:** 10.1002/fsn3.685

**Published:** 2018-07-04

**Authors:** Millicent G. Managa, Peter P. Tinyani, Grany M. Senyolo, Puffy Soundy, Yasmina Sultanbawa, Dharini Sivakumar

**Affiliations:** ^1^ Phytochemical Food Network Research Group Department of Crop Sciences Tshwane University of Technology Pretoria South Africa; ^2^ Queensland Alliance for Agriculture and Food Innovation Center for Food Science and Nutrition The University of Queensland Brisbane QLD Australia

**Keywords:** minerals, phytochemicals, postharvest chain, qualitative losses, vegetables

## Abstract

This study was initiated to investigate the impact of transportation, storage, and retail shelf conditions on lettuce quality and phytonutrients losses in the urban fresh produce market supply chain. Reducing postharvest losses is a priority to reduce the loss of the dietary‐based phytonutrients and to improve the health of the consumers. Limited information is available in South Africa related to the postharvest and nutrition loss in the urban fresh produce market supply chain. In this study, we quantified the postharvest losses, changes in phytochemicals, and loss of minerals in lettuce at different points of Tshwane Fresh Produce Market supply chain. Lettuce supply to the Tshwane Fresh Produce Market from two different provinces, Gauteng and North West, were included in this study for comparison. Lettuce from the two provinces was collected from five different farms. The loss of fresh weight, changes in visual quality, phytonutrition properties, and economic loss of lettuce at the supply chain points: (a) transport; (b) storage; (c) and at the retail shelf was investigated. Five boxes of lettuce per supply chain point from Gauteng and North West provinces were randomly selected. The results indicated that the high temperature (25°C) and low RH (40%) at the retail shelf affected the weight, overall quality, and phytonutrition properties of lettuce. Cumulative economic loss was higher at the retail shelf due to the inferior quality of lettuce. The study identified where major quality and phytonutrition losses occur during marketing. The study demonstrated to identify the where major food and nutritional loss losses occur during marketing. This information will significantly benefit food sustainability by introducing technologies to manage food and nutrition losses.

## 1 INTRODUCTION

The production of vegetables is constrained by postharvest losses, which in turn limit the volumes of good quality produce reaching consumers. Food losses and waste contribute to postharvest losses while the reduction of postharvest losses is reported as a critical component of ensuring future global food security (Aulakh, Regmi, Fulton, & Alexander, 2013). Postharvest losses are also associated with malnutrition (FAO, 2011). Therefore, to sustain food security, food availability needs to be increased via reduction of postharvest losses during the supply chain (Aulakh, Regmi, Fulton, & Alexander, 2013). At the same time, reducing food waste improves marketing and investment for the growers (Kader, 2005). Postharvest food losses include quantitative (decrease in weight) and qualitative (reduction or changes in nutritional values, taste, color, texture, or appearance) or economic losses (Buzby & Hyman, 2012).

Consumption of leafy vegetables such as lettuce is very popular nowadays not only due to their freshness but also for nutritive value and health benefits (Ansorena et al., 2012). Crisphead lettuce which is also known as Iceberg lettuce (*Lactuca sativa* L.) is considered to be one of the furthermost popular, economically important and commonly consumed vegetables. The health benefits of consuming lettuce on a daily basis are incontestable because lettuce constitutes a rich source of active bioactive compounds which include flavonoids, phenols, antioxidants, vitamins (C, A, and folate), and minerals (potassium, phosphorus, and calcium) (Cruz et al., 2014). Several major causes of the losses at the retail shelf include poor storage conditions, high temperatures, and rough handling of lettuce heads by market intermediaries and consumers (Kanlayanarat & Acedo, 2011). Insufficient pest control management and nutrient supply during pre‐harvest periods are also cited as exerting a negative impact on the postharvest quality of lettuce (Sharma & Singh, 2011). Frequently, such product that does not meet the required market standards and it is rejected at the market level (DAFF, 2016). Although the postharvest loss of vegetables has been assessed in certain reports in various countries, to our knowledge the impact of supply chain factors on the loss of bioactive compounds and minerals in lettuce was not studied in detail. In light of these considerations, the aims of the present study were (a) to quantify the loss and changes in predominant bioactive compounds such as phenolic acids (chlorogenic, chicoric, caftaric, and caffeic acid), flavonoids (quercetin), carotenoids, total chlorophyll, and ascorbic acid at different points (transport, storage, and the retail shelf) of the supply chain at Tshwane Fresh Produce Market; (b) to identify the supply chain points where the predominant losses occur; and (c) to do an economic analysis of postharvest losses of lettuce.

## 2 MATERIALS AND METHODS

### 2.1 Experimental plan and sampling

Five commercial farmers from Gauteng and North West provinces, who are regular suppliers of crisphead lettuce to the Tshwane Fresh Produce Market, were included in this study. These two provinces were selected because they fall among the top lettuce producers in South Africa, and they are the major lettuce suppliers to the Tshwane market throughout the year. Five replicate boxes each containing eight heads total weight of 5 kg of lettuce from each province (North West and Gauteng) were drawn randomly from each point of the supply chain: (a) after the transport (the transportation was done using a refrigerated truck at 10°C and 80% RH); (b) during storage at the market (fluctuated from 15 to 20°C); and (c) at the retail shelf for quantitative and qualitative loss assessments. A completely randomized design was adopted in this study. Thereafter, two heads of lettuce from each box were snap frozen in liquid nitrogen and held at −80°C to assess the changes in phytonutrition properties. Also, quantitative and quantitative loss and the changes in phytonutrition properties at the retail shelf from Day 1 to Day 4 were conducted to determine the saleable date of the lettuce to ensure that consumers purchase lettuce that is of good quality and high nutritional value.

### 2.2 Temperature and RH assessments

Temperature (°C) and RH (%) were recorded using a Tinytag T/RH data loggers (Gemini data loggers Ltd., UK) during storage and at the retail shelf.

### 2.3 Crisphead quality analysis

The percentage weight loss was determined by subtracting sample weights from their initial recorded weight and presented as a percentage of the initial weight (Aguero, Ponce, Moreira, & Roura, 2011). Lettuce head color was impartially measured with a Minolta CR‐400 chromameter (Minolta, Osaka, Japan). Color value measurements were taken at three main points on each head, from the outer part, the middle part, and the inner part of the lettuce head. The color changes of crisphead lettuce were quantified in the L*, a*, b*, C, and h° color values (Ren, Li, Qian, Fan, & Du, 2017). The quality defects yellowing, rusty brown, stem discoloration and wilting, and the overall acceptance (based on color, free from defects or decay) were evaluated and scored following a five structure hedonic rating scale (Zhan, Hub, Li, & Panga, 2012) where 5: severe, 4: moderately severe, 3: moderate, 2: slight, 1: fairly free, and 0: none. Overall acceptance rating of three was considered the acceptable limit for sale or consumption (Zhan et al., 2012).

### 2.4 Determination of bioactive compounds

Ascorbic acid content was determined using the 2,6‐dichlorophenolindophenol dye titrimetric method (AOAC International, 2002). For the determination of chlorophyll, carotenoids, phenolic acids, flavonoids (quercetin), antioxidant capacity, and scavenging activity leaf samples were snap frozen in liquid nitrogen and stored at −80°C to compare the variation in bioactive compounds during the different supply chain points operated in two different provinces.

Chlorophyll *a* (*Chl a*), chlorophyll *b* (*Chl b*), and carotenoid contents were determined as described by Mampholo, Sivakumar, Beukes, and van Rensburg (2013) where snap frozen lettuce head leaf samples of 0.02 g (five replicate samples per treatment) were mixed with 1 ml methanol and extracted for 2 hr. *Chl a* and *Chl b* contents were calculated using the following formula: *Chl a *=* *15.65 A_662_ − 7.340 A_646_, *Chl b *=* *27.05 A_646_ − 11.21 A_662_, total carotenoids = 1,000 A_470_ − 2.270 *Chl a* – 81.4 *Chl b*/227. The data were expressed as mg total chlorophyll and carotenoid as 100 g^−1^ FW.

For the quantification of phenolic acids (chlorogenic, chicoric, caftaric, and caffeic acid) and flavonoids, 5 g of snap frozen samples was extracted in 16 ml of methanol–water–formic acid mixture (25:24:3 v/v/v) using an ultrasonic extraction device as described previously (Malejane, Tinyani, Soundy, Sultanbawa, & Sivakumar, 2017; Ntsoane, Soundy, Jifon, & Sivakumar, 2016). Thereafter, extracted samples were centrifuged at 12,000 × *g*, 7 min, 10°C and the supernatant (35 μl) filtered using hydrophobic PTFE syringe filters (0.22 μm pore size). Ten microliters were injected thrice for high‐performance liquid chromatography (HPLC) (with a photo diode array ultraviolet detector, C18 column [100 × 4.6 mm; 5 μm particle size], Model Flexar ^™^ 89173‐556 PerkinElmer, Waltham, MA, USA). The mobile phase, flow rate, and gradient elution program were according to chromatogram that was read at 265, 280, and 320 nm. Phenolic acids were identified and quantified using pure external standards and constructing of calibration curves (Ntsoane et al., 2016). Results for phenolic acids were reported in mg per kg of fresh weight.

Total antioxidant capacities were determined using ferric reducing antioxidant power (FRAP) and 2,2‐azinobis (3‐ethyl‐benzothiazoline‐6sulfonic acid) (ABTS^+^) activity assay. The ferric reducing antioxidant potential (FRAP) assay was performed according to Malejane et al. (2017) and Llorach, Tomás‐Barberán, and Ferreres (2004) without modification using FRAP solution (0.3 mM sodium acetate [pH 3.6], 10 mM 2,4,6‐tri pyridyl‐2‐triazine [TPTZ], and 20 mM FeCl_3_ [10:1:1 v/v/v]). A fresh sample (5 g) was mixed with methanol: water (4:1 v/v). A FRAP solution (950 μl) at 37°C was mixed with 50 μl of the sample mixture. Trolox solution (10–250 mg/L) was used to construct the calibration curve for quantification of FRAP antioxidant activity. The results were expressed as kmol Trolox equivalents antioxidant capacity (TEAC) per kg fresh weight (kmol TEAC/kg).

The ABTS^+^ assay was performed using a method described by Arnao, Cano, and Acosta (2001). Samples of 0.2 g of lettuce (snap frozen sample) were extracted twice with 1.5 ml of 80% methanol. The stock solutions included 7.4 mM ABTS^+^ solution and 2.6 mM potassium persulfate solution. Thereafter, the working solution was prepared by mixing the two stock solutions in equal net and allowing them to react for 12 hr at room temperature in a dark place. The solution was then diluted by mixing 1 ml ABTS^+^ solution with 22.5 ml methanol to obtain an absorbance of 1.1 ± 0.002 units at 734 nm using a multiplate reader and ensuring that fresh ABTS^+^ solution was prepared for each assay. Lettuce extracts (15 μl) were allowed to react with 285 μl of the ABTS^+^ solution for 2 hr in a dark place and the absorbance was taken at 734 nm using a multiplate reader. Calibration curves were constructed for each assay using Trolox as the standard. The antioxidant activity (ABTS assay) was expressed as TEAC.

### 2.5 Mineral analysis

Lettuce leaves were oven dried at 70°C for 48 hr and thereafter ground and sieved to quantify the leaf N, P, K, Ca, Mg, Zn, and Fe content using the ICP OES (inductively coupled plasma optical emission spectrometry) according to Mahlangu, Maboko, Sivakumar, Soundy, and Jifon (2016).

### 2.6 Economic losses

Economic loss was calculated by computing the monetary value of the physical loss acquired by expressing the percentage of physical loss as a fraction of the actual selling price of each lettuce box per kilogram.

### 2.7 Statistical analysis

The experiment was conducted twice during 2016 and 2017 winter season and since there are no significant variations between the seasons (year), the data were pooled together for the statistical analysis.

All data were analyzed with the aid of the Genstat program version 15.1, and to determine the mean differences, Fisher’s protected least significant differences (LSD) at *p *<* *0.05 level of significance was performed.

## 3 RESULTS AND DISCUSSION

### 3.1 Temperature, RH fluctuation, and weight loss

Cumulative temperature and RH measurements taken during storage and the retail shelf for four consecutive days are shown in Table 1. The overall temperatures and the RH at the storage point varied between 4.94–6.39°C and 62.2% and 66.6%, respectively. However, the overall temperature and RH varied between 21.4–24.2°C and 25.6%–39.9%, respectively at the market shelf (Table 1). Temperature and RH are major environmental factors that are crucial in maintaining quality and extending the shelf life of fresh fruit and vegetables (Kitinoja, 2002). The total weight loss of crisphead lettuce at three stages of the supply chain within two provinces exhibited great variation with produce accumulating the greatest weight loss at the retail shelf (Table 2). Significant weight loss (45.31%) occurred at the retail shelf (Day 3) than in storage where the weight loss was 25.55%, while the produce after transportation did not show any weight loss (0.00%). However, the magnitude of weight loss of lettuce failed to differ significantly between the two provinces (Table 2). Weight loss increased significantly with increasing time (days) at the retail shelf and on Day 4 the weight loss was 64.6% (Table 3). It is evident from these results that weight loss is the most important cause of postharvest deterioration because it is associated with the saleable weight. The observed fluctuation between the temperature and the RH (Table 1) is responsible for the observed weigh loss in this study.

**Table 1:** Temperature and RH in storage and at the retail shelf at the Tshwane Fresh Produce Market

Supply chain points	Day 1		Day 2		Day 3		Day 4	
	Temperature (°C)	Relative humidity (%)	Temperature (°C)	Relative humidity (%)	Temperature (°C)	Relative humidity (%)	Temperature (°C)	Relative humidity (%)
At storage	5.8 ± 0.8	64. ± 3.0	6.3 ± 9.2	62.2 ± 0.0	6.0 ± 2.4	65.8 ± 0.0	4.9 ± 3.8	66.6 ± 0.0
At the retail shelf	24.2 ± 0.1	37. ± 2.0	24.0 ± 3.7	25.6 ± 0.0	21.4 ± 6.0	39.9 ± 0.0	23.0 ± 7.9	29.9 ± 00

**Table 2:** Variation in quality attributes of Crisphead lettuce at the different supply chain points of the Tshwane Fresh Produce market

Supply chain point	Weight loss (%)	Yellowing (scale)	Rusty brown (scale)	Stem end discoloration (scale)	Wilting (scale)	Overall acceptance
Transport	0.10c ± 2.30	0.8c ± 1.20	1.9c ± 1.31	2.30c ± 1.31	0.65c ± 1.24	1.43a ± 1.34
Storage	25.55b ± 1.31	1.6b ± 1.10	3.3b ± 1.26	3.47b ± 1.08	1.54b ± 1.31	2.49b ± 0.76
Retail shelf (Day 3)	45.31a ± 2.40	2.0a ± 1.45	4.4a ± 1.53	4.49a ± 110	2.39a ± 1.56	3.02c ± 1.81
Province						
GP	20.05^ns^ ± 1.67	1.30^ns^ ± 1.60^.^	3.00^ns^ ± 1.05	3.30^ns^ ± 1.56	1.36^ns^ ± 1.12	2.20^ns^ ± 1.10
NW	27.13 ± 1.20	1.70 ± 3.02	3.40 ± 1.14	3.54 ± 1.67	1.70 ± 1.65	2.42 ± 1.56

*Note*

Means followed by the same letter within the column are not significantly different, *p *<* *0.05.

**Table 3:** Variation in ascorbic acid content and visual quality parameters of Crisphead lettuce obtained from two provinces at the retail shelf of Tshwane Fresh Produce Market

Treatment	Ascorbic acid (mg 100 g^−1^FW)	Weight loss (%)	Yellowing (scale)	Rusty brown (scale)	Rib discoloration (scale)	Wilting (scale)	Decay (scale)
Provinces							
GP	39.71^ns^ ± 0.45	27.10^ns^ ± 1.76	1.96^ns^ ± 1.98	3.4^ns^ ± 1.87	3.7^ns^ ± 1.21	1.6^ns^ ± 1.2	0.5^ns^ ± 1.23
NW	34.00 ± 0.52	30.07 ± 1.65	2.00 ± 1.67	3.6 ± 1.01	3.6 ± 1.86	1.8 ± 1.5	0.6 ± 1.98
Retailers shelf (Days)							
Day 1	54.40a ± 0.85	1.20d ± 2.10	0.8d ± 1.23	1.7d ± 1.23	2.2c ± 1.34	0.9c ± 1.31	0.1c ± 1.23
Day 2	47.85b ± 0.72	8.08c ± 2.14	1.7c ± 1.56	2.4c ± 1.87	3.6b ± 1.67	1.5b ± 2.54	0.4b ± 1.12
Day 3	28.30c ± 0.92	45.31b ± 2.40	2.0b ± 1.45	4.2b ± 1.53	4.5a ± 1.10	2.4a ± 1.56	0.5b ± 1.05
Day 4	16.87d ± 0.81	64.59a ± 1.86	3.3a ± 1.35	5.0a ± 1.75	5.0a ± 1.56	4.7a ± 1.91	1.3a ± 1.31

*Note*

GP: Gauteng province; NW: North West province.

Means followed by the same letter within the column are not significantly different, *p* < 0.05.

### 3.2 Quality losses

Quality losses due to yellowing, rusty brown, stem discoloration, and wilting damage were significantly higher in lettuce at the retail shelf (Table 2). Also, there were no significant differences observed between the two locations where the lettuce was obtained with respect to the above‐mentioned quality defects (Table 2). Rusty brown and stem discoloration slightly increased during storage (3 days), but the quality was still regarded as acceptable for sale and consumption according to the hedonic scale less than 3 (Table 2). At the retail shelf (Day 3), browning and rib discoloration increased severely exhibiting a hedonic scale more or less 3 and the product and shows limited marketability became unmarketable (Table 2). However on Days 3 and 4, the visual quality decreased due to increased browning, pinking, and wilting exceeding the hedonic scale score of 4. It is interesting to note that on Day 3 and Day 4, the lettuce heads at retail shelf the average losses for ascorbic acid were 48% and 68.9%, respectively (Table 3). The major reason for wilting was water loss due to transpiration through the stomata when the concentration gradient of the water vapor between the air spaces in the leaf and the surrounding environment was steeper (lower RH) (Ben‐Yehoshua & Rodov, 2003). Furthermore, fluctuation of temperatures (high) or RH (low) was shown to affect the quality of leafy vegetables (Ansorena et al., 2012; Munhuweyi, Linus Opara, & Sigge, 2016). Water loss results in cell wall degradation and affects the texture, turbidity and visual appearance (Ansorena et al., 2012; Piagentini, Güemes, & Pirovani, 2002), loss of vigor, and wilting (de Oliveira, Leal, Honório, & Soare, 2013).

The L* (lightness) color value describes the brightness of the leaves. Among the three points of the supply chain, lettuce heads withdrawn during transport showed the highest L* value expressing the brightness of the leaves, whereas lettuce heads at points of storage and the retail shelf demonstrated lower brightness (Table 4). Significant change (lower) in *h°* in lettuce heads withdrawn at the retail shelf indicated that lettuce heads were becoming more yellow in color (Table 4). The color coordinate a* showed a higher negative value in lettuce heads withdrawn at the transport point and it decreased significantly at the retail shelf indicating the onset of browning (Table 4). The significant decrease in color coordinate b* value in lettuce from the retail self was mainly as a consequence of higher browning (Table 4) which can be related to the production of browning pigment due to the activity of polyphenol oxidase enzymes (Tomás‐Barberán, Gil, Castañer, Artés, & Saltveit, 1997).

**Table 4:** Variation in color values of Crisphead lettuce at different supply chain points of the Tshwane Fresh Produce market

Supply chain point	L*	a*	b*	*h*°
Transport	60.69a ± 3.43	−11.0a ± 2.16	18.13b ± 2.87	119.6a ± 3.63
Storage	54.60b ± 2.34	−9.83b ± 2.76	16.46c ± 1.56	118.5b ± 2.75
Retail shelf (Day 3)	47.18c ± 2.65	−9.24c ± 3.01	15.52 ± 2.67	117.4c ± 2.67
Province				
GP	58.70^ns^ ± 2.10	−9.76^ns^ ± 2.56	18.48^ns^ ± 3.52	117.8^ns^ ± 2.43
NW	49.61 ± 3.45	−10.31 ± 3.12	18.72 ± 2.76	119.1 ± 2.45

*Note*

GP: Gauteng province; NW: North West province.

Means followed by the same letter within the column are not significantly different, *p* < 0.05.

There were no significant interaction for color values L*, a*, b*, and *h°* in lettuce withdrawn at the different points of the supply chain from different provinces (Table 4). The observed temperature and the RH (Table 1) are responsible for the observed changes in color coordinates. It also taken into consideration that the freshness and lightness or brightness (L*) of the product is another attribute that attracts the consumer (de Oliveira et al., 2013).

### 3.3 Variation in bioactive compounds

A significant decrease in ascorbic acid, total chlorophyll content, and carotenoids was noted in the lettuce heads withdrawn at the retail shelf point (Table 5). The ascorbic acid content was 54.91 mg 100 g/FW after transport and 25% of the ascorbic acid content in lettuce was lost after storage. Moreover, approximately 48% of the ascorbic acid content was lost on the retail shelf during marketing (Day 3). Similarly, total chlorophyll was reduced at the storage point and at the retail shelf by 38.2% and 67.3%, respectively. Influence of temperature on color and chlorophyll degradation was shown by Cantwell and Kasmire (2002). Loss of chlorophyll in the leaves leads to increase the intensity of yellow color in the leaves and yellowing of leafy vegetables is associated with the activity of peroxidase and lipoxygenase. Chlorophyll is responsible for the green color and is used as one of the quality parameters that determine the consumer purchasing power (de Oliveira et al., 2013). Loss of chlorophyll content at the retail shelf affected consumer preference for purchasing lettuce and influenced the marketing of lettuce in this study. Loss of chlorophyll is primarily due to the senescence that is associated with loss of membrane lipids and proteins, thus leading to textural changes and cell death (Page, Griffiths, & Buchanan‐Wollaston, 2001). Loss of ascorbic acid content from the outer and middle lettuce leaves was related to its reduced moisture content caused as a result of lower RH and the higher temperatures at the retail shelf (Marfil, Santos, & Telis, 2008). Ascorbic acid content was reported to improve the lightness of strawberries, peaches, and apples (Rababah, Ereifej, & Howard, 2005). Previous reports stated that 5%–12% loss of ascorbic acid occurs at 30 and 40°C, respectively, during the 24 hr delay between harvesting and processing (Kader & Morris, 1978). The daily requirement of ascorbic content for a man is 90 mg/d and for women, 75 mg/d (Frei & Trabe, 2001). The study clearly demonstrated that around 81% of the ascorbic acid content was lost on the retail shelf on the Day 4 (Table 3). In the present study, the similar reduction trends were observed with light intensity (L* values) and the ascorbic acid content of lettuce at the retail shelf (Tables 2 and 3). Presence of higher ascorbic acid content was reported to reduce browning and it acts as an anti‐browning agent (Landi, Degl’Innocenti, Guglielminetti, & Guidi, 2013). The observed decline in ascorbic acid content at the retail shelf also explains the increased browning (rusty browning and rib discoloration in lettuce in this study Table 3). The observed loss of visual quality is on the other hand associated with the loss of ascorbic acid that acts as an anti‐browning agent and also due to the increase activity of the PPO activity (Tomás‐Barberán et al., 1997).

**Table 5:** Variation of ascorbic acid, quercetin, total chlorophyll, carotenoids, and antioxidant properties of Crisphead lettuce at the different supply chain points of Tshwane Fresh Produce Market

Supply chain point	Ascorbic acid (mg 100 g/FW^−1^)	Quercetin (mg GAE 100 g/FW^−1^)	Chlorophyll (mg 100 g /FW^−1^)	Carotenoids (mg 100 g/FW^−1^)	FRAP (mg 100 g/FW^−1^)	ABTS (mg 100 g/FW^−1^)
Transport	54.91a ± 0.89	3.62c ± 0.69	6.73a ± 0.56	5.94a ± 0.53	0.209c ± 0.95	−0.311c ± 0.68
Storage	41.20b ± 0.67	7.15b ± 1.10	4.16b ± 0.627	4.58b ± 0.63	0.497b ± 0.62	1.094b ± 0.75
Retailers shelf (Day 3)	28.30c ± 0.56	11.78a ± 0.45	2.20c ± 0.73	3.10c ± 0.75	1.071a ± 0.74	1.784a ± 0.58
Province						
GP	48.68^ns^ ± 0.76	6.83^ns^ ± 0.87	5.94^ns^ ± 0.95	4.47^ns^ ± 0.68	0.539^ns^ ± 0.1.2	0.641^ns^ ± 1.23
NW	34.26 ± 0.94	7.20 ± 0.71	2.79 ± 0.84	4.61 ± 1.58	0.646 ± 0.83	1.069 ± 0.96

Note. GP: Gauteng province; NW: North West province.

Means followed by the same letter within the column are not significantly different, *p *<* *0.05.

The total carotenoid content was also reduced by 22.9% and 47.8% at the storage point and the retail shelf, respectively (Table 5). Furthermore, loss of carotenoids was significantly higher at 25°C in leafy vegetables subjected to higher temperatures during thermal treatments, which have been reported to induce loss of carotenoids in vegetables (Nyaura, Sila, & Owino, 2014). The findings of Nyaura et al. (2014) concur with the decrease in total carotenoid compounds in lettuce at the retail shelf at the Tshwane Fresh Produce Market (Table 5).

An increasing trend was noted in quercetin content, the antioxidant capacity FRAP, and the ABTS^+^ in lettuce heads at the retail shelf (Table 5). However, the observed variation in ascorbic acid, total chlorophyll content, carotenoids, quercetin content, and the antioxidant capacity (FRAP and the ABTS^+^) in lettuce heads withdrawn at different points of the supply chain from the two provinces (Gauteng × North West) were not significant (Table 5). Crisphead lettuce contained higher levels of chicoric, caftaric, and chlorogenic acids than the other phenolic acids (Table 6). The levels of phenolic acids significantly increased after transport and remained at higher concentrations at the retail shelf (Table 5). Abiotic stress conditions, including temperature and RH variation, often favor the accumulation of flavonoids (Dixon & Paiva, 1995). The observed increase in the concentration of quercetin, phenolic acids (chicoric and chlorogenic acids), and antioxidant activity at the retail shelf could be due to temperature and RH fluctuations (Tables 5 and 6). Chicoric and chlorogenic acids act as substrates for enzymatic browning or pinking in lettuce (Luna et al., 2012; Monaghan, Vickers, Grove, & Beacham, 2017). However, in this study, observed increase in phenolic acid at the retail shelf was more less similar to the observation reported by Zhao, Carey, Young, and Wang (2007). The phenylalanine ammonia‐lyase activity could have increased in response to microbial decay, mechanical damage (Cantos, Espín, & Tomás‐Barberán, 2001) and could have resulted in an increased biosynthesis of phenolics during storage and at the retail shelf. On the other hand, the increased concentration of phenolic acids (Table 6) also confirms that there is another mechanism involved in lettuce browning other than the PPO activity as suggested by Mai and Glomb (2013).

**Table 6:** Variation of phenolic acids (chicoric, caffeic, caftaric, and chlorogenic acids (mg g FW^−1^) of Crisphead lettuce at the different supply chain points of Tshwane Fresh Produce Market

Supply chain points	Chicoric acid	Caffeic acid	Caftaric acid	Chlorogenic acid
Transport	0.43b ± 0.45	0.16b ± 0.71	0.28b ± 0.61	0.20b ± 0.19
Storage	0.59b ± 0.032	0.25a ± 0.13	0.39b ± 0.36	0.30b ± 0.42
Retail shelf (Day 3)	0.94a ± 0.65	0.39a ± 0.23	0.60a ± 0.84	0.53a ± 0.64
Provinces				
GP	0.39b ± 0.94	0.15b ± 0.51	0.28b ± 0.21	0.13b ± 0.29
NW	0.92a ± 0.58	0.38a ± 0.73	0.57a ± 0.92	0.55a ± 0.68

*Note*

GP: Gauteng province; NW: North West province.

Means followed by the same letter within the column are not significantly different, *p *<* *0.05.

The increase in phenolic acids was responsible for observed higher antioxidant power (FRAP) and the antioxidant capacity (ABTS^+^) (Table 5). Correlation between the phenolic compounds and FRAP or ABTS^+^ has been established (Wootton‐Beard, Moran, & Ryan, 2011). Therefore, the unmarketable lettuce can be freeze dried and used as functional food ingredient for food supplementation programs.

### 3.4 Loss of mineral elements

Loss of mineral elements such as K, P, Ca, Mg, Fe, Zn, Mn, Cu, and B was significantly higher at the retail shelf (Table 7A,B) and there were no significant differences observed between the crisphead lettuce obtained from the two locations (Gauteng and North West). The reduction of K, P, Ca, and Mg at the retail shelf was 23.5%, 21.3%, 24.8%, and 14.3%, respectively (Table 7A).

**Table 7:** (A) Loss of major mineral elements in Crispy head lettuce at different points of the supply chain of Tshwane Fresh Produce Market. (B) Loss of trace mineral elements in Crispy head lettuce at different points of the supply chain

(A)								
Supply chain points	K		P		Ca		Mg	
	(mg/kg)	% reduction	(mg/kg)	% reduction	(mg/kg)	% reduction	(mg/kg)	% reduction
Transport	35,913a ± 2.23		5,400a ± 2.34		5,280a ± 1.34		2,030a ± 0.75	
Storage	33,552a ± 1.64	−6.57	5,330a ± 0.84	−1.3	4,965a ± 0.96	−5.96	1,890b ± 1.46	−6.89
Retail shelf (Day 3)	27,468b ± 0.97	−23.5	4,250b ± 1.58	−21.29	3,970b ± 1.65	−24.81	1,740c ± 0.75	−14.28

(B)										
Supply chain points	Fe		Zn		Mn		Cu		B	
	(mg/kg)	% reduction	(mg/kg)	% reduction	(mg/kg)	% reduction	(mg/kg)	% reduction	(mg/kg)	% reduction
Transport	1,870a ± 01.64		155a ± 0.85		96.5a ± 0.96		43.5a ± 0.73		51.7a ± 0.85	
Storage	958b ± 0.854	−48.77	128b ± 0.64	−17.41	51.4b ± 0.74	−46.73	23.3b ± 0.84	−46.43	46.4b ± 0.5.3	−10.25
Retail shelf (Day 3)	773c ± 0.94	−58.66	102c ± 0.56	−34.19	48.5b ± 0.83	−49.74	8.8c ± 0.65	−79.77	39.8c ± 0.61	−23.01

Note. Means followed by the same letter within the column are not significantly different, *p *<* *0.05.

The levels of K, P, and Ca in lettuce heads did not vary significantly between the transport and storage points (Table 7A). The trace mineral elements such as Fe, Zn, Mn, Cu, and B were reduced by 48.8% 34.0%, 49.7%, 79.8%, and 23.0% at the retail shelf in this study (Table 7B). The loss of mineral elements at the retail shelf was also reported in leafy vegetables (Gogoa, Opiyob, Ulrichs, & Huyskens‐Keila, 2017). The loss of mineral elements in this study was mainly due to the higher temperature at the retail shelf that had favored the metabolic changes and senescence (Gogoa et al., 2017). Also the minerals such as iron and calcium in vegetables can chemically altered and interact with other compounds (Buescher, Howard, & Dexter, 1999). Sometimes, the storage conditions can influence the oxidation state of minerals (Fe and Cu) that can reduce the solubility and the bioavailability of these minerals (Buescher et al., 1999). The loss of ascorbic acid during storage could have affected the iron availability (Frossard, Bucher, Machler, Mozafar, & Hurrell, 2000) in the lettuce in this study.

### 3.5 Economic losses

The cumulative losses at the storage and the retail shelf (Day 2) were 36.7% and 53.1%, respectively (Table 8A). The total economic losses at storage and retail shelf were ZR 144 and ZR 146 (Table 7A). On average, 119 boxes of crisphead lettuce were delivered from Gauteng Province farms on Day 1 and 46 boxes were sold and 72 boxes were left on the retail shelf (Table 8B). Each box was sold at a price of R 56/box, whereas the revenue of lettuce heads sold on Day 1 was R 2,622 and the market agent commission of 7% was R 196.65. Of the 72 boxes left on Day 1, 47 boxes were sold on Day 2 at a price of R 36/box. However, 25 boxes were not sold. On Day 2, there was a slight decrease in revenue R2116 as well as the agent market commission (R158.7). Of the lettuce heads that were not sold on Day 25 boxes were sold at a reduced price of R 22/box on Day 3. There was a drastic decline in the revenue (R 416) as well as the market agent commission (R 31.2). On Day 4, the price of the unsold produce was reduced again to R 14/box with 7 boxes left unsold at the retail shelf. It is evident from this study that the economic loss of Crisphead lettuce during supply chain was higher at the retail shelf (Table 8B).

**Table 8:** (A) Economic loss of Crisphead lettuce during supply chain points at the Tshwane Fresh Produce Market. (B) Postharvest losses on quantity delivered, revenue, and market agent commission

(A)				
Supply chain points	Final fresh weight Box kg^−1^	Loss kg^−1^	Cumulative produce loss (%)	Economic loss (ZR)
Transport	4.9	—	—	—
Storage	3.1	1.8	36.7	144
Retailers shelf (Day 3)	2.3	2.6	53.1	146

(B)						
	Quantity delivered on the retail shelf (Boxes)	Quantity Sold (Boxes)	Quantity remaining on the retail shelf (Boxes)	Price (R/Box)	Revenue (R)	Agent commission 7.5%
Retail shelf (Days)						
Day 1	118.6	46.2	72.4	56	2,622	196.65
Day 2	72.4	46.6	24.8	36	2,116	158.7
Day 3	24.8	17.8	7	22	416	31.2
Day 4	7	0	7	14	0	0

The geographic site of production, distance from the Tshwane Fresh Produce Market, conditions during agricultural production, postharvest handling, and storage conditions at the farm could have played a vital role in determining the quality (Gogoa et al., 2017). It is evident in this study that economic losses associated with crisphead lettuce increased along the supply chain. Although these varied between the provinces, the cumulative losses (R/kg) were higher at the retail shelf (Table 7), where the price value was determined by the weight loss and overall quality (Gogoa et al., 2017).

This study illustrated the impact of temperature accumulated at the retail shelf depends on the duration (number of days) (Hertog, Lammertyn, Scheerlinck, & Nicolaï, 2007) and it is evident in this study that the impact of accumulated temperature difference significantly affected the visual quality and the nutritional value with an increase in the number of days. In addition, this study further showed the importance of maintaining the cold chain on the retail shelf to reduce the 45% weight loss of crisphead lettuce in this study. Also, this type of study can be adopted for different commodities and the information generated will benefit in improving the cold chain technologies to reduce food loss and nutrition.

## CONFLICT OF INTEREST

Non conflict of interest between the authors.

## ETHICAL STATEMENT

This study does not include any animal or human testing or questionnaire analysis on market survey.
